# ‘Two-faces’ of hyaluronan, a dynamic barometer of disease progression in tumor microenvironment

**DOI:** 10.1007/s12672-023-00618-1

**Published:** 2023-01-25

**Authors:** Ying Liu, Li Li, Li Wang, Lu Lu, Ying Li, Guolin Huang, Jinjing Song

**Affiliations:** 1 Department of Pharmacology, Guangxi Institute of Chinese Medicine & Pharmaceutical Science, Nanning, 530001 Guangxi People’s Republic of China; 2 Guangxi Key Laboratory of Traditional Chinese Medicine Quality Standards, Guangxi Institute of Chinese Medicine & Pharmaceutical Science, Nanning, 530001 Guangxi People’s Republic of China; 3 School of Medicine & Health, Guangxi Vocational & Technical Institute of Industry, Nanning, 530001 Guangxi People’s Republic of China; 4 Department of Pharmacy, Guangxi Orthopaedics and Traumatology Hospital, Nanning, 530012 Guangxi People’s Republic of China; 5 Department of Pharmacy, The First People’s Hospital of Nanning, Nanning, 530022 Guangxi People’s Republic of China

**Keywords:** Hyaluronan, Extracellular matrix, Hyaluronan catabolism, Tumor microenvironment, Extracellular signal

## Abstract

Hyaluronan (HA) is a linear polysaccharide consisting of disaccharide units which are the d-glucuronic acid and n-acetyl-d-glucosamine. As the largest component of the extracellular matrix in microenvironment, HA polymers with different molecular weights vary in properties to molecular biology function. High molecular weight HA (HMW-HA) is mainly found in normal tissue or physiological condition, and exhibits lubrication and protection properties due to its good water retention and viscoelasticity. On the other hand, an increase in HA catabolism leads to the accumulation of low molecular weight HA (LMW-HA) under pathological circumstances such as inflammation, pre-cancerous and tumor microenvironment. LMW-HA acts as extracellular signals to enhance tumorigenic and metastatic phenotype, such as energy reprogramming, angiogenesis and extracellular matrix (ECM) remodeling. This review discusses the basic properties of this simplest carbohydrate molecule in ECM with enormous potential, and its regulatory role between tumorigenesis and microenvironmental homeostasis. The extensive discoveries of the mechanisms underlying the roles of HA in various physiological and pathological processes would provide more information for future research in the fields of biomimetic materials, pharmaceutical and clinical applications.

## Introduction

Cancer diseases have long been regarded as the most threatening burden of human healthcare. Being the largest populated country, annual cancer incidence in China contributed to nearly 50% of the total global cancer incidence, meanwhile, cancer mortality sustained at the highest level (over 50%) worldwide [1, 2]. Since the GLOBOCAN project was held in the 1980s, cancer incidence and mortality increased with breast, lung, colorectal, gastric and prostate accounting for over 50% of all cancer types [3, 4]. Those cancers normally induce latent symptoms that alarm patients when they undergo a late stage. Terminal stage of cancer mostly developed metastasis which gradually leads to multiple organ failure. Researchers found that the metastasis progression of tumors obeyed the “seed and soil” theory with partial phenotype transformation [5]. In micro-perspective principles of the tumor environment, different tumor cells required specific “soil” for their prosperity [6].

The tumor microenvironment (TME) is the local biological environment in which the tumor cells live. Microenvironment homeostasis depends on a complicated and dynamic compensation mechanism [7]. It is currently known that the tumor microenvironment contains a large amount of extracellular matrix (ECM), the main components of which include collagen, non-collagen glycoproteins, glycosaminoglycans and proteoglycans. Changes in the composition and total content of ECM can strongly affect the biological properties of tumor cells and stromal cells, such as proliferation and motility, and play an important role in tumor metastasis [8]. Hyaluronan (HA) is the main component of glycosaminoglycans in ECM, accounting for about 85% of ECM components [9]. As the largest component of ECM, hyaluronan (HA) acted as a supportive framework in tumor stroma [10, 11]. Normally, homeostasis of HA stayed at the dynamic balance between biosynthesis and hydrolysis every second accompanied by a fluctuant distribution of HA with different molecular weights during either physiological or pathological processes [12].

Under physiological conditions, HMW-HA, synergistically produced by cell membrane HA synthetase (HAS) as tail elongation in a “pendulum” manner, occupies the interstitial space among multiple organs [13]. Pathological conditions such as tumors, inflammation, oxidative stress, and tissue remodeling exacerbate HA degradation, which rapidly degraded into small fragment of different length (molecule weight ranging from 1–1000 kDa) by hyaluronidases (hyaluronidases, HYALs) [14]. Therefore, the fast degradation sometimes is incomplete resulting in the accumulation of low-molecular-weight products of HA in the interstitial space of organs or tissues lesion (arthritis and tumor) [15, 16]. Interestingly, LMW-HA or small HA fragments, but not HMW-HA, are critical for malignant progressions such as tumor invasion and metastasis. Here, we described the turnover and catabolism of HA under different conditions and discussed the causes of tumorigenesis and microenvironment homeostasis regulated by those different types of HA.

## HA distribution and molecule weight composition

HA is composed of disaccharide units with β-1,4 or β-1,3 glycosidic bonds and stabilized as linear polymer glycosaminoglycan with molecule weight ranging from 10^3^ to 10^7^ kD [17, 18], depending on the length of disaccharide units. HA can be classified as high or mega-molecule weight HA (HMW-HA or vHMW-HA), with over 1000 kDa; medium molecular weight HA (MMW-HA) with 100 ~ 1000 kDa, low molecular weight HA (LMW-HA) with 20-100 kDa, and HA fragment (fgHA) from oligosaccharide to 20 kDa [19, 20]. In different circumstances, the dominant HA could be very different among microenvironment, since the varied molecular weight HA displays exclusive work from physiological protection to oncogene promotion, depending on the stage of the disease.

In physiological conditions, the highest HA concentration is present in synovial fluid reaching around 1000 μg/mL, followed by that in the vitreous body (around 680-1800 μg/mL), cumulus cell–oocyte complex (100-500 μg/mL), dermis (200 μg/mL), brain (30-120 μg/mL) and the skin (17.5–23.5 μg/mL) [21–25]. HMW-HA has strong water retention and swelling properties, and its volume can be extended up to 1000 times its original size after absorbing water, forming a complex network structure to accommodate the liquid molecules. Therefore, HA is a biological "lubricant" involved in lubricating joints or holding gelatinous connective tissue together [9]. HA fills among soft connective tissues of organs, keeping moisture retention and protecting joint cartilage from mechanical friction. Peri-cellular HA absorbs water from interstitial space, where the mesh scaffolds were formed to expend a moisture living space, tightly surrounding as a hydrogel halo which avoided tissue compression from growth [26].

The catabolism of HA slows down in lymphatic systems, thereby the HA level is stabilized at a certain level. In lymphatic systems, the concentration of HA ranges from 0.5–18 μg/mL, wherein the highest level of HA in thoracic lymph reached 8.5 μg/mL for adults *vs* 18 μg/mL for children [27]. However, circulated HA in blood stays low in concentration (below 1 μg/mL) [28]. The low concentration of HA might be attributed to the fast uptake by the liver or endothelial cells, yielding a large amount of turnover about 10–100 mg per day and a fast half-time of degradation (2–5 min) in blood [27–29]. According to this physiological property, the increase of serum HA attributes to pathophysiologic deposition of a large amount of HA, leading to circulating accumulation. Serum HA level now is widely recommended as an indicator of liver fibrosis, a vital early sign of liver cirrhosis [30–32]. Compared to the normal control, the mean value of serum HA level has tenfold high in patients with chronic hepatitis, 15-fold higher in patients with liver cirrhosis, but only 5 times that in patients with liver cancer liver [33]. Also, over-produced HA from active HA synthesis in the liver activates the hepatic stellate cells resulting in liver fibrosis [34]. Besides, the decreased concentration of HA in arthrosis is a vital index of patients with rheumatic arthritis (RA) and osteoarthritis (OA) wherein the overloaded inflammation pathogenesis caused hyper-degradation of HA [35].

## Property and variety of HA under different microenvironment

In the physiological aqueous solution, polymers of HA self-assemble H-bonds with solvent to generate many randomly hydrophobic zones being regarded as ‘tangle’ of HA cross-link [36]. These hydrophobic pockets endow HA aqueous the property of non-Newtonian flow, bearing elasticity, viscosity and water retention [37]. These structures however are loose and deformed but easily recovered, since the H-bonds only consist of weak non-covalence. Among the microenvironment, various glycoproteins attach to the liner chain of HMW-HA with non-covalent bonds, which stabilizes their location and buffers them in the ECM of organs. Besides, under the catalysis of TSG-6, HMW-HA covalently binds to the heavy chain of IαI which is recruited by tetramer pentraxins to block the amplification of inflammation [38, 39]. On the other hand, this property makes HA a good lubricant in the joint cavities or buffer zones between tissues once the microenvironment shear stress increases and interstitial space decreased [40]. In the tumor microenvironment, LMW-HA takes a high proportion because the balance of biosynthesis and degradation has been interrupted, resulting in incomplete digestion of HMW-HA to LMW-HA or fgHA. The solution of LMW-HA still bears part of viscoelasticity, in a wide range concentration of 10–20,000 μg/mL [36].

In cosmetic use, HA filler is predominant the consumables. During the aging process, a large amount of HMW-HA or vHMW-HA is underwent depolymerization in skin tissues, which attributes to the accumulation of cellular metabolites. The injection of cosmetic HA filler, normally makes of HMW-HA or vHMW-HA, can postpone this cell aging progression in the skin [41]. Evidence in rodent models illustrated that older mice contained lower HA amounts than their younger counterparts [42]. The decreased HA was found in damaged skin from UV-irradiation and photo-damaged conditions. When treating mechanical damage of the cartilago with intra-articular injection of HA filler, symptoms would be released due to recovery of the lubrication within the cartilage [43, 44]. Based on the good swelling property and water retentivity without immune rejection, HA filler or cross-linked HA hydrogels have been widely used as anti-aging and anti-wrinkle products in terms of slowing down HA degradation through HMW-HA overload [45].

Under the physical condition, LMW-HA is rather taken up by phospholipids endocytosis and is hydrolyzed into UDP-oligosaccharide in the lysosome. However, in pathological situations, an increase of LMW-HA attributed to active hyaluronidase or accumulated free radicals would be a marker of disease exacerbation such as inflammatory filtration, tissue injury and metastasis [46, 47]. LMW-HA loses lubrication function since the deficiency of hydrophobic interspaces causes a decrease in viscoelasticity leading to increase tissue rigidity [28]. Besides, high LMW-HA inclines chronic inflammation which induced the initiation of irreversible diseases such as cartilage damage, fibrosis and pre-cancerous condition [48–50].

### HA bio-synthesis

The synthesis of HA could be briefly introduced as HAS recruited and connected UDP-sugars by forming β-glycosidic bonds at the cellular membranes associated with energy consumption. The major site of HA biosynthesis is derived from fibroblasts of tumor stroma rather than epithelium, wherein the HASes are highly expressed [51, 52]. Since HASes family are all anchored in the plasma membrane with an active region located inside the cytoplasm. HA, therefore, was formed intra-membrane [53]. Before the start of synthesis, HAS requires a lipid environment wherein the combination of cardiolipin and active site triggered its activity, while the binding of cholesterol attenuated its activation [54]. Another step would be the recruitment of two UDP-sugar substrates, UDP-GlcNAc and UDP-GlcUA, and two catalytic glycosyltransferases [54]. When biosynthesis is initiated, a chitin primer containing hexosaminidase with 6 constant GlcNAc work as a leading signal [55]. Due to the steric hindrance between UDP-GlcNAc and UDP-GlcUA, the ‘Pendulum Model’ was introduced by P. H. Weigel as the putatively dynamic progression. When the reducing end addition of HA chain elongation occurred, the chemical structure of both GlcUA-β(1,3)-GlcNAc bond and GlcNAc-β(1,4)-GlcUA-bond limited formation of long conjugation system thereby alternatively forming non-planar conformation of the chain [54].

**Fig. 1:**
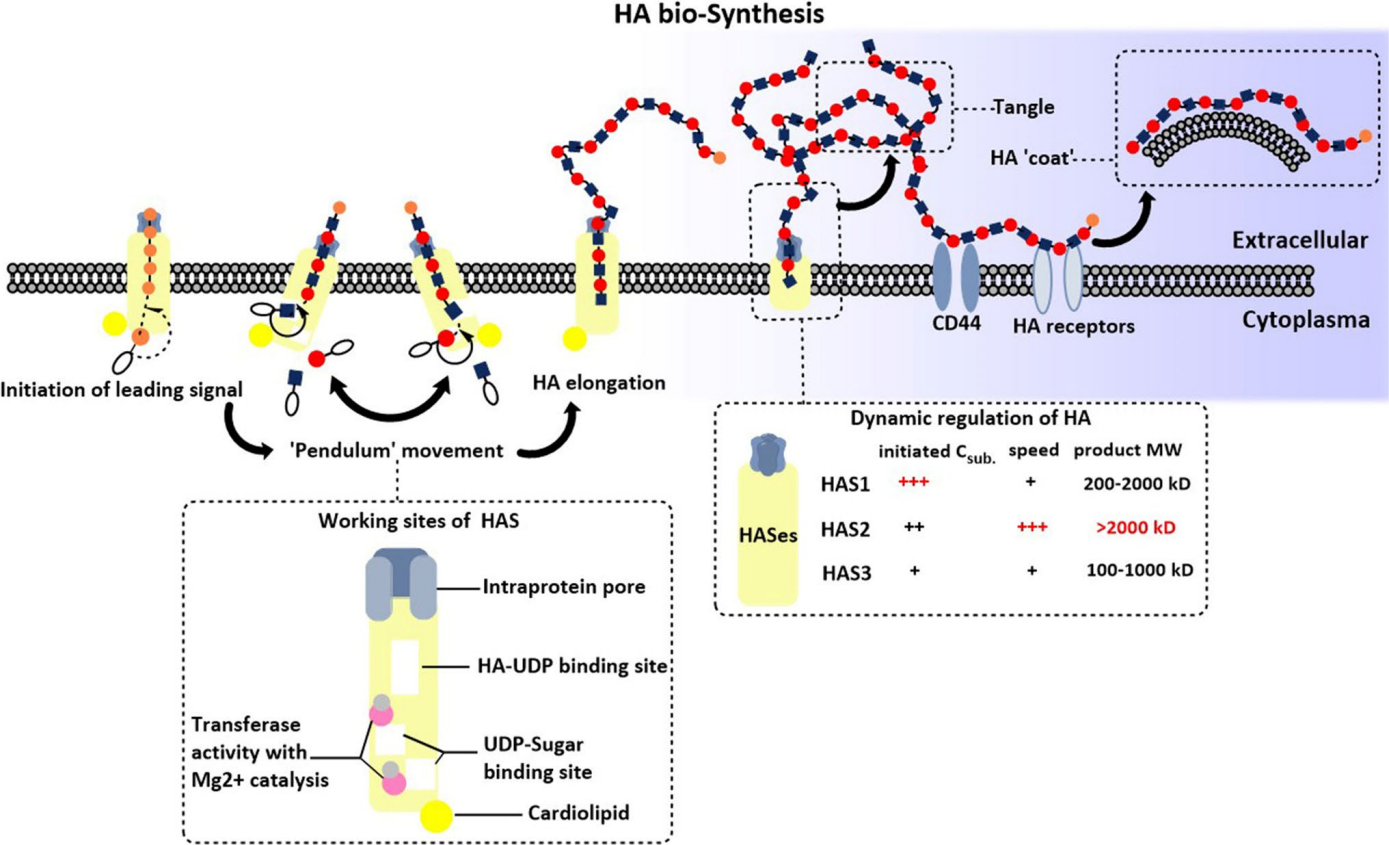
The bio-synthesis of HA. The process of HA synthesis in the ‘Pendulum’ movement pathway starts from the initiation of the leading signal, a chitin primer connecting 6 constant GlcNAc. Two substrates of UDP-sugars, UDP-GlcNAc and UDP-GlcUA were recruited to the membrane, and generated linear polymers connecting with GlcUA-(1,3)-GlcNAc -β(1,4)-GlcUA-bond. The residue addition synthesis was produced inside the HAS, and was delivered by transferase under Mg^2+^ catalysis. The HA polymers were extruded through intra-protein pore of HAS, hanging out of the extracellular space nearby the membrane or folding as tangle through the intra-molecular and intermolecular H-bonds. Different HA receptors recognized specific HA polymers with different molecule weights. For example, CD44 captured HMW-HA and formed HA ‘coat’ on the external surface of cells. The lower left box with dash line indicates the detail and specific position of HAS how it produces the HA synthesis. The lower right box with the dash line indicates the working requirement and products of HA synthesis when three HAS isoforms, HAS1, HAS2, HAS3, start dynamic regulation

There were two theories explaining HA excretion, one of them was a pressure-regulated molecule pump that recognized intracellular HA and excrete extracellular matrix [53]. The synthesized HA does not remain with HAS for a long time due to the Brownian movement. Along with HA addition, a higher pressure would be gained. Therefore, the outflow of HA was essential for releasing intra-protein pressure as well as lengthening the elongation reaction. The other theory preferred that HA was slowly extruded in situ through the protein pore when the chain reaches ~ 20 kDa [56]. The HA chain outflow might loosely attach to the extra-membrane region of HASes, forming the HAS-HA complex. The accumulation of this complex remains the key regulator for slowing down HA synthesis (Fig. 1).

**Fig. 2:**
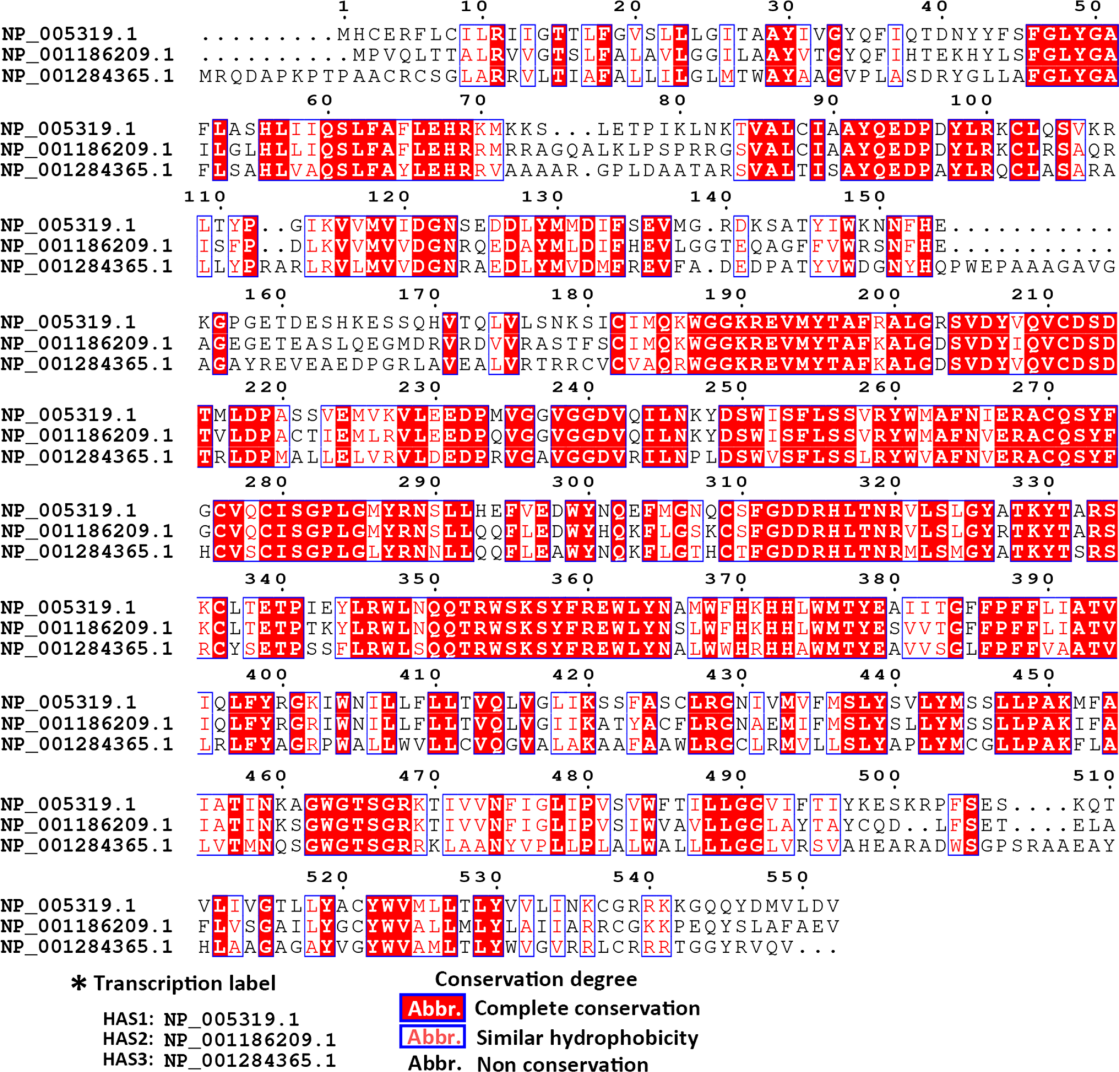
The amino acid sequence alignment of three hyaluronan synthases. CLUSTAL X was used for analyzing the multiple alignments of the amino acid sequence of HAS1, HAS2, HAS3. Then the result of alignment was visualized by ESPript 3.0. The transcript label listed on the left was the code from NCBI database of the amino acid sequence of HAS1, HAS2, and HAS3, respectively. The amino acid abbreviation in white color with red background presents the aligned sequences are identical, and words in red color present the aligned sequences have similar hydrophobicity, hydrophilicity or similar side chain. Word in black presents the aligned sequences that are not conservation

Active and adequate HASes kept HA at the required level. Although the fast turnover of stromal HA causes HA insufficiency, tumor cells may compensate for the expression and activity of HASes at this moment. Tumor cells, normal and cancer-associated fibroblasts could generate HA, though the type and amount of HA vary in specific circumstances [57, 58]. Although the three HAS subtypes had 55% identical sequence to each other (Fig. 2), each of them has the difference in affinity and catalytic efficacy with UDP-sugar substrates, which was determined by the Km value, obeying the Michaelis–Menten equation. The initiation and amount of HA synthesis depend on the intracellular concentration of UDP-substrate and the expression and activity of HAS [59, 60]. HAS1 obtained the highest Michaelis Km value which reflected the lower affinity to substrates and slower rate for HA formation [61]. HAS3 could be activated at very low substrate content and the increase in substrate level affects the elevated synthetic amount of HA. Unlike HAS3, the HA production by HAS1 would only be started when the UDP-sugar doubled compared with the other two isoforms However, HA production generated by HAS1 remains the most sensitive to UDP-sugar changes. HAS2 produced the largest amount of HA, and the initiation threshold of HA generation only required low UDP-sugar. Besides, the synthetase activity of HAS2 is less sensitive to the rise of substrate level [60]. Thus, the microenvironment HA was mainly generated by HAS2, which requires lower activation energy and faster synthetic speed. HAS2 focus on recruiting MMH-HA to produce vHMW-HA while HAS1 and 3 contribute to MMH- to HMW-HA (with molecular weight ranging around 200kD-2000kD and 100-1000kD), respectively [62]. After extracellular emission of HA, long polymer HA tangled to net structure that could let it stably wrap at the cell surface. HA net works as a sponge, whereby providing the lubrication and supporting effort between cell–cell interaction [63]. Therefore, the over-expression of HAS2 is more meaningful among multiple biological progressions.

Additionally, HAS2 predominates the synthesis of HA in early embryonic development. In the mice model, the absence of HAS1 and HAS3 does not affect the growth or fertility of mice. Importantly, embryonic development is severely damaged in the HAS2 deletion mice model, and the lack of HAS2 reduces cell migration and epithelial-mesenchymal transformation, failing to differentiate mature cells [64]. Aside from generating HA, HAS could act as prognosis indicators in cancer diseases. In the MDA-MB-231-BM cell, high expression of HAS2 promotes invasion, leading to the occurrence of bone metastasis. On the contrary, the transfection of HAS2 siRNA can inhibit the development of breast cancer and the occurrence of metastatic colonization through the EGF-mediated kinase/PI3K/Akt signalling pathway [65]. In contrast, suppression of HAS2 by using antisense-transcript HAS2-AS1 has been shown to attenuate the proliferation and malignant phenotype of glioma, osteosarcoma and breast cancer [66–68]. Furthermore, in the analysis of gene expression profiles of pancreatic cancer cells and fibroblasts, HAS2 was highly expressed as a differential expression gene and was associated with increased invasiveness of pancreatic cancer cells [69]. Similarly, high expression of HAS1 and HAS3 either tumor tissue or tumor stroma predicts poor prognosis or malignant progression in breast cancer, lung cancer, bladder cancer, and also chondrosarcoma [70–73].

### HA bio-hydrolysis

Nature balance could not permit infinite HA synthesis. When the threshold of ambient pressure is affected by the increase of HA content, both HAS activity and HA emission will be halted and the switching of HA hydrolysis will be launched. All biological changes require balance, and accumulation of HA in physiological situations accompanied by the acceleration of HA degradation. This process includes the recognition of HA, hyaluronidase involvement and optimal physicochemical property. HYAL 1–5 and PH-20 (coded by SPAM1) were the major enzymes that could degrade HA into fragments [74, 75].

**Fig. 3:**
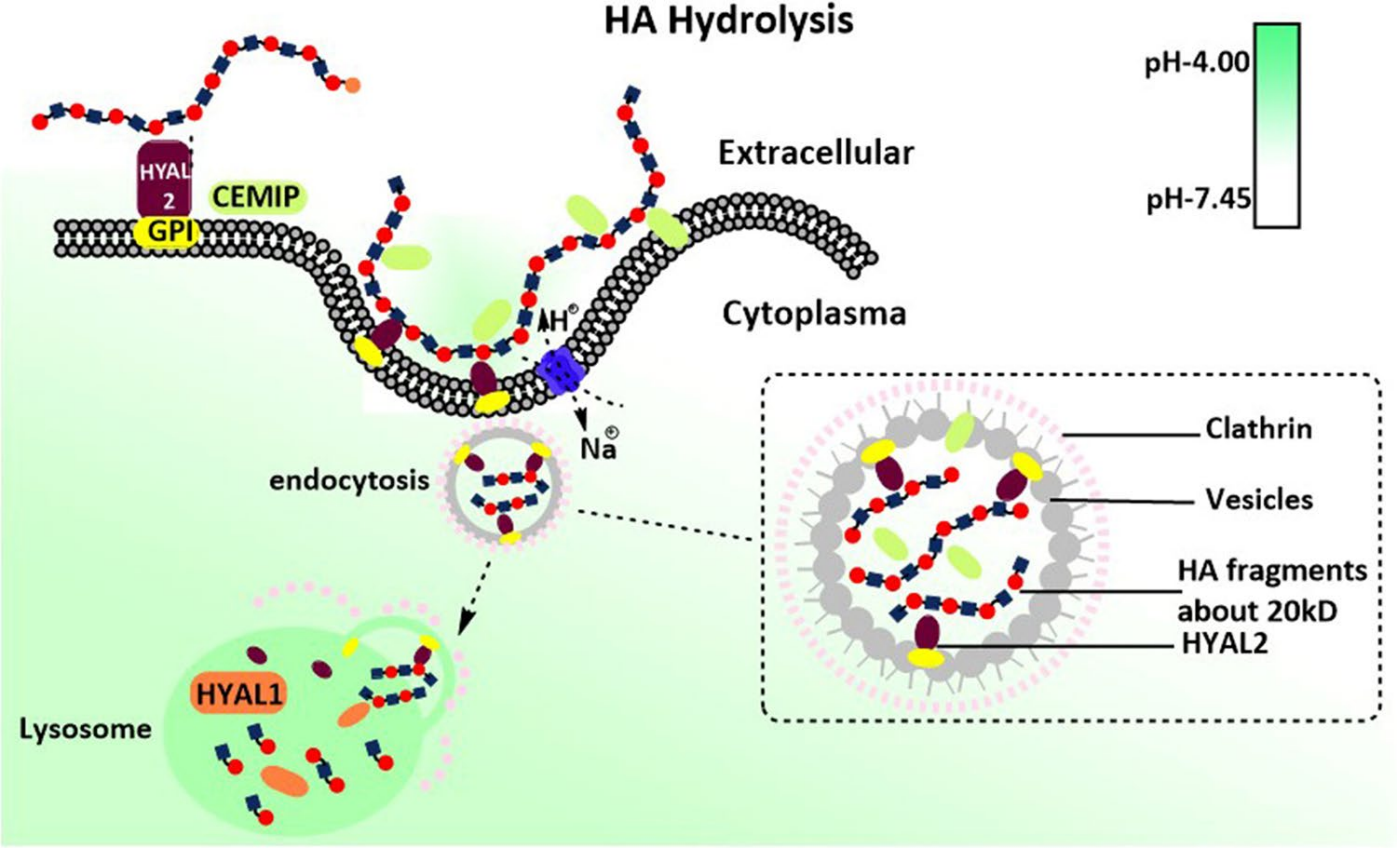
The hydrolysis of HA. The two-step process of HA hydrolysis. The first step happens in the ECM, HA is caught by the membrane enzyme HYAL2, which is anchored by GPI and exposed to the external surface. Or like CEMIP, which attached membrane or secreted into ECM. HA and enzymes then form a complex to initiate the endocytosis process. And then, clathrin is recruited at the internal face of invagination at the membrane. During this progression, Na^+^-H^+^ ion pump promotes ion exchange for providing energy for endocytosis and adjusting local pH for optimum enzyme activity. HA is digested to LMW-HA (about 20kD) in vesicles, which are encapsulated by clathrin. After being delivered to the lysosome, endocytosis vesicles release content into the lysosome followed by enabling the second step. In the lysosome, HYAL1 take over the final process that cut off LMW-HA into smaller fragments or oligosaccharides. HYAL2 shows no more enzyme activity since the pH in lysosome let it inactivate. These oligosaccharides later may either enter the metabolic pathways or reassemble with UDP and be synthesized to HA again

The distribution of HYALs varies in different organs. HYAL1 and HYAL2 are widely detected in many organs except the brain in which HYAL2 has not been detected [76]. HYAL3 and PH-20, on the other hand, are abundant in specific parts [63]. Since HYAL4 shows no degradation activity of HA while HYAL5 did not express in the human body, these two enzymes would not be further discussed in this review. Major functional enzymes HYAL1 and HYAL2 are located at the lysosomes and plasma membrane respectively. To be specific, the 448 glycines of HYAL2 is attached to the glycosylphosphatidyl inositol (GPI) resulting in its membrane location [76]. HA degradation relies on two steps (Fig. 3), the polymers were recognized by HA receptors and HYAL2, covering the outer face of the membrane. And then HA/HYAL2 forms complex which is cupped by the membrane to form caveolae. This invagination process is associated with the activation of Na^+^-H^+^ exchange protein that triggered the inflow of H^+^ [10]. The Na^+^-H^+^ exchange and electrochemical flow that come from this ion channel also take responsibility for energy supplement for HA synthesis from the hydrolysis of UDP-sugar bond, instead of directly applying high-energy phosphate bond from ATP or GTP [54]. As a result, the optimal acidic environment is achieved where the pH level is lower than 4, thereby initiating the HA hydrolysis reaction. At this step, the minimum HA fragment remains 20kD regardless of C4 or C3 connective glycosidic linkage, as the HYAL2 endowed both hyaluronan glucuronidase and hyaluronan glucosaminidase activity. When the endocytosis of HA fragments are being carried out with vesicle coating chatinin, this will be delivered to the lysosomes to start the final step of HA degradation [10].

Lysosomes is the major and final hydrolysis place wherein HYAL1 sustains highly activated Both HYAL1 and HYAL2 require a specific pH environment to gain the best active state. Under the optimum pH, HYAL1 merges endocytosis to digest MMW-HA or intermediates HA to yield HA fragments as small as 6 glycosamine units. In fact, HA with 20 kDa molecular weight does not bind HAYL1 and HYAL2 easily, even under the optimal acidic environment. Besides, HYAL2 has a relatively lower affinity with it, therefore, HYAL1 takes over the rest of the hydrolysis process [35]. Recently, another protein named CEMIP is suggested as a novel hyaluronidase that endowed similar hydrolysis ability as HYAL2, but independently bound to CD44 [10]. CEMIP is encoded by the gene KIAA1199, which is located at chromosome 15q25.1 [77], whereas the other coding gene of HYALs is located at chromosome 3. The location of CEMIP mainly anchors in the rough endoplasmic reticulum, also distributed among nuclear, cytoplasm, membrane and extracellular matrix [77]. The hydrolyzed function of CEMIP depends on the active domain at N-tenimus, where is also the signal domain of cytoplasm secretion, as researchers summarized before [77–79]. No reports provided the direct interaction process between CEMIP and HA, though one of the research groups first proposed that membrane CEMIP could direct connect HA without interacting with CD44 by adding purified recombinant CEMIP into HMW-[^3^H]HA tracking with ^3^H label [35]. Many pieces of research indicated the indirect depolymerization of CEMIP on HA by setting up a CEMIP-efficient model in multiple cell lines and CEMIP knock-out transgenic animals [80]. However, the over-expression of CEMIP correlated to HA degradation frequently associated with increased protein amount of HYAL2, this evidence implied the interaction of CEMIP and HYAL2 might be complementary.

## Connecting microenvironmental signal to intracellular signalling, HA receptors

As a polysaccharide, HA alone does not modulate cellular biological changes but along with intermediary tools, such as HA receptors to deliver different messages. HA receptors receive various signals and stimulate both inhibitory and oncogenetic signalling depending on HA species. According to the current studies, there are 7 HA receptors, CD44, RHAMM, TLR2, TLR4, LYVE, LAYN and STAB2, which participate in connecting extracellular signal and intracellular signalling in tumor diseases.

### HMW-HA/receptors complex system induces tumor suppression effects

For example, in the physiological or precancerous condition, HMW-HA acts as the protective coat surrounding the cell surface, which is recognized by representative HA receptors such as CD44 and RHAMM, forming loose bonds and delivering chemical signals towards hyaluronidase (HYALs) to sustain the stable proliferative rate of normal cells [10, 81]. Even in the pro-metastatic stage, HMW-HA plays major roles in anti-angiogenesis, facilitating anoikis, impeding proliferation, and invasion [20, 40, 82–84]. Sliver et al. reported that HMW-HA inhibited tumor cell uptake of nutrients and angiogenesis through CD44-mediated integrin protein attachment and hydration pathways [85]. The good affinity of HA and CD44 allows them to form a tight HA-CD44 complex with a coat form surrounding membrane. According to this property, remolding intracellular CD44 into soluble CD44 in breast cancer dramatically reduces the binding amount of HMW-HA with CD44 in the cancer cell membrane, blocking HA-mediated intracellular signal transduction, and promoting cancer cell apoptosis, cycle arrest and other phenotypes [86, 87]. In addition, increasing the secretion of soluble CD44 in lung metastases of breast cancer can stimulate the interstitial tissue to produce a large amount of HMW-HA, forming a physical barrier and inhibiting the colonization of metastatic cells [86]. Besides, when HMW-HA binds to LYVE-1, the permeability and integration of lymphatic endothelial cells are broken down in tumor tissues [88, 89].

### LMW-HA/receptors complex system facilitates tumor progression

Nevertheless, LMW-HA could also bind to the receptors such as CD44 and LYVE-1 but exhibited the opposite effects [90]. LMW-HA induces the secretion of inflammatory factors, matrix hydrolases and pro-angiogenic factors by activating the specific receptors such as TLR2, TLR4, and LAYN of tumor cells, which dominates in the field of metastasis and colonization [82, 91]. After LMW-HA binds to LAYN, inflammatory infiltration in the microenvironment is attribute to the activation of NF-κB signalling under the over-production of MMP-1 and MMP-13 [92]. The binding of LMW-HA to the receptor LYVE-1 activates the transcriptional activity of the β-catenin protein, destroying the integrity of the lymphatic endothelium and promoting lymphatic metastasis in melanoma [93]. Among many kinds of LMW-HA, LMW-HA with the molecular weight of less than 10 kDa is more likely to bind to TLR2. Scheibner et al. found that in injured lung tissue, when TLR2 recognizes LMW-HA, its intracellular MYD88 and TRAF-6-dependent signalling pathways are activated, resulting in the promotion of the migration of lung cells to finish damaged repairs [94]. In addition, genetic down-regulating TLR4\TLR2 expression inhibits M1 macrophage phenotype induced by LMW-HA and the stimulation of TLR4/MYD88 after LMW-HA activation generated more inflammatory factors PEG2 in macrophage cells [90]. Capturing and directing immune cell migration attributed to the formation of receptors-HA-receptors (CD44 and LYVE-1) sandwiches. Immune cells such as dendritic cells (DCs), whose membrane anchored CD44 with high density, are wrapped by LMW-HA forming a CD44/HA coated surface. When these coated cells travel in vessel system, LYVE-1 in the outer layer of the lymphatic vessel seizes the HA shell which firstly attaches to the membrane of ECs followed by either passing through the gap junction between cells or accessing the lumen of vessels [89, 95].

As for the MMW-HA, this segment was regarded as transient intermediates, they either undergo re-uptake by synthases for chain elongation or be invaginated by membrane HYALs for lysosome hydrolysis [96]. When HYALs decomposed the small HA fragment, the oligosaccharide is drawn into the glycol-metabolism cycle and reassembled to synthetic substrates. Due to the situation of transition and pathologic homeostasis, HA fragment with a wide range of molecular weight co-existed in ECM along with disease progression.

## Genetic susceptibility of HA-enzymes

Tumourigenesis attributes to various genetic mutations, one of the important mutation types is the deletion of the coding region in chromosomes which is highly related to the occurrence of tumor [97]. Apart from all types of tumors, there is a major susceptible deletion of hyaluronidases whose coding sequence located in chromosome 3p21.3 across ethnics and countries that contributed to lung cancer [97–102]. Major mutations of various 3p regions found in many cohorts of LC were the deletion of which the sequence of hyaluronidase (HYAL) 1 2 and 3 was located [103–105]. HYAL1 and 2 participate in the separation part of HA degradation, whereas HYAL3 barely exerts hydrolase function but sustains the pluripotency of stem cells [106]. Compared to HYAL3 which contains a highly conserved sequence, large-scale SNP cohorts indicated that mutation happens more often in HYAL1 and HYAL2 [107, 108]. The other mutation is due to the single nucleotide polymorphism which resulted in the change of coding amino acid, leading to loss of hydrolase activity. Other than ‘have or not’ regulation in DNA level, quantity regulation in transcription and protein levels are also crucial. A great deal of research in the past few years has focused on the post-translational modification and epigenetic control of the HASes enzymes. The concentration of HA is related to distortions, while the number and size of HA are related to the alternating expression and activity of HASes and HYALs [109]. HASes deficiency is an autosomal recessive genetic disorder [110].

## Pre-metastatic barometer in the tumor microenvironment

### Being a prognosis indicator in clinical application

Despite that healthy homeostasis requires enough HMW-HA level, LMW-HA maintained at a high level further enables inflammatory attack and it was also maintained in tumor microenvironment turning up either in peri-tumoral stroma or intra-tumor [111–113]. Evidence showed that enrichment of stroma HA is prevalent among many types of tumors, including bladder, ovarian, breast, prostate, pancreatic, colon and lung cancer, indicating a high possibility of tumor invasion and metastasis [9, 114–118]. This phenomenon mostly accorded to poor survival and advanced tumor stage thereby high HA status becoming a poor prognostic factor for tumors. In the research in the field of ovarian cancer, the stromal HA level positively correlates to advanced progression since the metastatic sites have higher HA filtration [119]. In addition, higher stromal HA indicates a patient with an aggressive stage and a bad outcome in breast cancer. Moreover, high intra-stroma HA levels are also correlated to deep filtration and distant metastasis in colon cancer [120]. Similarly, high HA level could not only be detected in the stroma of prostate cancer, but also the intra-tumor accumulation [121]. This is due to influx uptake or self-generation through tumor cells. High levels of serum LMW-HA are positively correlated with breast cancer lymph node metastasis and the invasive potential of breast cancer cells, whereas reducing LMW-HA production can significantly inhibit the migration and invasion of breast cancer cells[46]. For example, LMW-HA can stimulate pancreatic cancer cell motility [122, 123].

If HA acted as a protective barrier of normal tissues, why is its accumulation surrounding the tumor microenvironment reflected to poor prognosis? This controversy might attribute to the application of specific HA binding antibody which is used as quantitative analysis rather than distinguishing the actual molecule weights of HA. Despite the high amount of HA accumulates in both normal and tumor tissues, the opposite effect might be due to the different molecule weight of HA. The HA recognition mainly depends on the length of N-terminal peptides, thereby the existence of HA with more than 2kD could be linked tidily. However, different molecular-weight-HA have specific roles in tumourigenesis. Once the vHMW-HA is broken, fgHA exerts oncogenetic promotion in aspect of tumor metastasis and metabolism [74]. The fgHA ranging from 20 to 200kD predominately functioned as an angiogenic modulator that stimulated endothelial cells migrated, preparing a supply network for tumor metastasis [124]. HA with 200-400kD molecular weight might be the hydrolysis intermediates, which are associated with tumor cell migration and anoikis resistance, although these intermediates would later be delivered into lysosome digestion. The other feature of fgHA (below 20kD) would be interrupting the interaction of HWM-HA-HA receptor–binding instead of fgHA-HA receptor-binding, followed by triggering cleavage of HA receptors [125]. The accumulation of HA intermediates elevated the average molecule weight of HA in microenvironment, so that interrupting the HA homeostasis by accelerating the synthesis of HMW-HA as well as the rate of HYAL1-degradation. These phenomena might explain why the observation of high HA level in different situated microenvironment displayed both oncogenic and suppressive functions.

### Being a regulator in EMT and wound healing progression

Epithelial-mesenchymal transition (EMT) marks one of the metastatic-driven factors and adaptive change. Since detaching from ECM and cell–cell connection triggers the programmed cell death of epithelial cells, the first step of metastatic tumor cells is activating the EMT process [126]. Due to the tight interaction of cells and ECM, HA also participates in the modulation of EMT in tumor cells. Taking breast cancer as an instance, at the primary lesion, pre-metastatic tumor cells undergo interstitial transition by increasing the expression of CD44S isoform, to which HA specifically binds to protect cells from anoikis [127]. HA also acts as an ECM signal to induce the nuclear translocation of CD44, thereby over-expressing lysyl oxidase through activating its transcriptional promoter. The increased expression of lysyl oxidase then facilitates Twist1 to finish the EMT progression [128]. In addition, Excessive production of HA by HAS2 induces over-expression of TGF-β which activates transcriptional factors Snail and Twist to initiate the EMT process in breast cancer cells. This transition progression lets those cancer cells regain stemness and into a higher malignancy [129]. LMW-HA (< 30kD) could increase the mRNA expression of a mesenchymal marker, Snail2 and Vimentin, while the MMW-HA(> 200kD) obviously increased the expression of the epithelial marker, E-cadherin [130].

HA also serves as a wound healing regulator in non-tumor diseases. Treatment of complications in Diabetics with HMW-HA hydrogel effectively reduces inflammation by recruiting M2 macrophages and mobilizing the M2-transition of M1 macrophages to the wound site [131]. Many hydrogels for treating skin wound healing contain HA, which needs to be modified or crosslinked to gelate before medication application [132]. In a German report on the treatment of chronic wounds, wound dressings containing HA exhibit a better cost-effective tendency considering the ratio of cure proportion to cost input. [133]. In summary, the promotion of wound healing in tissues relies on the increased swelling and elasticoviscosity property of crosslinked HA hydrogel, wherein HA works as a structural frame. However, HA plays a signal role to facilitate the EMT progression of tumor cells in the tumor microenvironment, wherein HA binds its receptor first and then activates the downstream triggers.

### Being a regulator in fuel recycling and supply

**Fig. 4:**
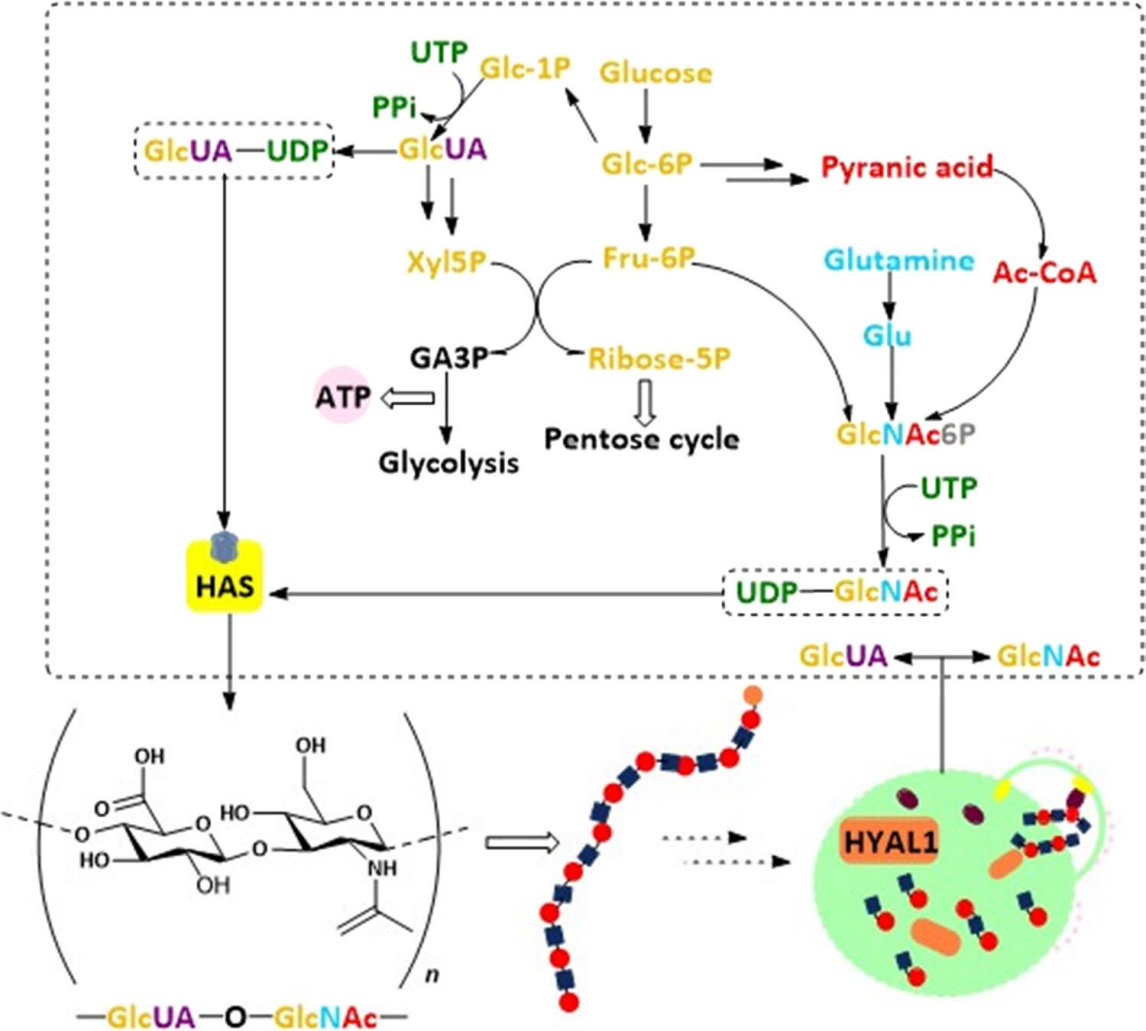
The energy metabolism of HA. The origins and decomposition of 2 substrates, GlcNAc and GlcUA, respectively. The ingredients consist of GlcNAc come glucose, glutamine and pyranic acid, and then UTP provides energy and attaches to GlcNAc. The glycosyl structure in GlcUA also comes from glucose as GlcNAc. In addition, GlcUA could also generate energy by entering glycolysis and the pentose cycle. The residues with different color track their source. The lower left displays a single unit of HA with a disaccharide structure. The lower right briefly flashback the final step of hydrolysis in lysosome where recycles GlcNAc and GlcUA to the cytoplasm

The catabolism of HA reprograms energy supply since it takes part in both consuming and generating energy through multiple metabolism pathways (Fig. 4). In human placenta-derived mesenchymal stem cells, HA at around 1 μg/mL acts as an inducer that switches on mitochondria function to enhance the glycolysis progression with about twice the amount of ATP and lactate production [134]. Radioactive element ^14^C was used to label HA, oral or intravenous administration in male SD rats. Ninety percent of exogenous intake HA was slowly absorbed from the digestive tract into circulation and then move to organs as part of the energy or took part in constituting to organ tissues. HA with labeling ^14^C was only found in the skin of the rats, while a large amount of ^14^C was detected in mice excrement [135].

Since the three major materials of GlcNAc are from the products of the hexosamine pathway, which connects carbohydrate, glutamine and nucleotide metabolism together, GlcNAc not only acts as a fundamental substrate of HA, but also a ‘sensor’ of energy availability in tumor cells [136]. Besides, GlcNAc takes over the glycosylation modification of protein, which draws much attention to the regulation of tumor phenotype [137]. Before GlcNAc or GlcUA consumes UTP to UDP-sugar, these two oligosaccharides enter the redox reactions associated with hexokinase and glucuronate reductase, respectively [112, 138]. Despite that tumor cells endow the ‘Warburg effect’, a different metabolism programming, rather than normal cells, degradation of HA is also joined to glycolysis and pentose pathway [138]. Once the energy supply becomes deficient, the “starved” tumor cells activate the secretion of the MMPs family, which cut the subtypes of collagen into pieces, so that they could be swallowed through pinocytosis. Following a similar pathway as collagen, the tumor ‘swallows’ HA or its fragment by endocytosis for energy replenishment [139–141]. The way endocytosis by cells relies on surface receptors of HA such as CD44, LYVE-1, HARE and RHAMM which can trigger HA uptake through self-assembling or recruiting clathrin-coated pits [35, 112]. This phenomenon was also observed in endocardial endothelial cells, wherein HA endocytosis works as high-energy metabolites to fuel cells [142]. Recently, a researcher reported that an increase of GlcNAc from HA cleavage remarkably ensures the energy supply in growth of pancreatic cancer cells [143].

Other evidence indirectly points out that the change of HA content or concentration modulated by alteration of HASes or HYALs also affects the energy supply. Glycolysis activity significantly increases in cells or xenografts when treated with hyaluronidase. It is mainly via ZFP36, the mRNA decay factor, which targets TXNIP transcripts to increase the internalization of the glucose transporter 1 (GLUT1) [144]. In triple-negative breast cancer, the expression of various enzymes involved in the glucose and glutamine metabolism showed obviously changed in the Hs578T subgroup which carries endogenous ^high^HA [145]. Also, the depletion of UDP-sugars dramatically inhibits the EMT progression, probably through reprogramming glycometabolism in breast cancer cells [146]. So far, there are limited studies on whether HA could be taken up by mitochondria as a substrate fuel, but ongoing insight has suggested HA metabolism might be a new bypass energy cycle in tumor disease.

### Being a regulator in angiogenesis balance

In a normal microenvironment, HMW-HA takes the major proportion which exerts anti-angiogenesis function. Because of the high hydration, HMW-HA is maintained in the interstitial space between endotheliocyte (ECs) [147]. Crowding these HMW-HA increased the permeation pressure forming physical barriers that restricted tumor invadesome entered circulation [148]. This physical barrier alternatively resisted inflammation infiltration and suppressed immune reaction [83]. Treating HMW-HA in cells causes the decreased expression of COX, and minimizes ROS through self-sacrificing electrons from glucosidic bonds [149]. Recently, a research team finds out that HMW-HA exclusively facilitates the angiogenic action of breast cancer by modeling its immune microenvironment. HMW-HA promotes the migration of ECs by stimulating monocyte/macrophagocyte to secret an increased amount of angiogenic factors, although a similar action is not observed in other cancers [150, 151] The infiltration and migration of monocyte/macrophagocyte are otherwise inhibited by increased concentration of HMW-HA in spinal nerves, maybe because HMW-HA competitive binds to receptors which block the activation of pro-inflammatory LMW-HA/receptor [152]. Based on current evidence, HMW-HA exerts anti-angiogenesis regulation among a majority of tumors, but still exhibits angiogenic action exclusively to specific one.

Contrarily, small HA fragments (~ 20 kDa) can stimulate neo-vascularization and promote tumor cell motility and invasion [153]. LMW-HA and fgHA stimulated angiogenesis in stimulating the growth and migration of ECs under hyper-inflammation [154]. On the surface of ECs, inflammatory signalling receptor TLR was triggered followed by emission of IL-6 and pro-inflammation factors IL-1β thereby boosting vasculum sprout [155]. Moreover, the accumulation of fgHA enhanced stromal VEGF levels, which specifically fostered vessel formation through binding to VEGFR of ECs [156, 157]. Besides, membrane receptor CD44 would transmit signals to NF-κB and TGF-βsignalling pathways [158, 159]. Once the LMW-HA binds to CD44, it would trigger the proliferation of endothelial cells through activating MAPK/SRC cascades [89, 160]. Another vital remodeling factor metalloprotease (MMPs) was also under HA regulation. High fgHA level promoted expression of MMPs such as MMP-13 and MMP9. When fgHA formed covalent bonds to HA receptors, the HMW-HA lost the possibility to compete for this target [161]. In summary, HA stimulated angiogenesis basically relied on indirectly affecting the proliferation and migration pathway of ECs. Different angiogenesis functions might be due to the chemical structure, since fgHA has centralized electron density that binds tightly to a single pocket of the receptor, while HMW-HA with nomadic polymer that formed multiple targeted sites of the receptor, thereby launching an opposite signalling pathway.

### Being a regulator in stiffness of cells and ECM

The change of HA either in content or molecular weight affects stiffness in a reciprocal way under the specific condition. On the one hand, accumulation of HA (HMW-HA and fgHA) assists collagen re-polymerization and secretion of integrin to increase ECM stiffness reaching 2–20 fold higher [162, 163]. Under this condition, the HA scaffold becomes denser by the bridge of integrin, and collagen filling enhances the structure. In cancer stroma, an increase of ECM stiffness leads to the formation of the porous net in tumor microenvironment [164]. Kaukonen R et al. reported the growth and invasion of breast tumor cells were inhibited in the normal stroma matrix while the inhibition was relieved under the tumor mimic matrix, which was stiffer than its counterpart [165]. Rising ECM stiffness fostered invasion and migration of breast cancer cells through translocating TWIST1 into nuclear, thereby promoting cellular transcriptional activity [166]. On the other hand, the replacement of glycosaminoglycans by HMW-HA without an additional cross-linker did not change the stiffness of ECM in glioma [167, 168].

Furthermore, the presence of HA in the medium could up-regulate intracellular stiffness, which increases cell movement and proliferation. These biological changes are similar to that of the tumors cultivated in stiffen matrix [167]. Higher HMW-HA density without cross-linker cause cellular stiffness instead of microenvironment stiffness might be due to the encapsulating membrane HA led to over-activate HA receptors. Due to the excellent biocompatibility and reciprocal plasticity, HA recently was utilized for constructing 3D scaffolds with varying stiffness to simulate tumor microenvironment [169]. They followed the principle of augmenting stiffness through increasing HA content and cross-link reagents to adjust suitable porous channels which approached to real microenvironment as much as possible.

### Being biomimetic materials in medical application

Recently, the application of HA coating capsules is beneficial for modifying drug delivery systems. In pre-clinical research, HA-coated sh-vector presented a better targeting ability in nude mice growing subcutaneous tumors. Research demonstrated that HMW-HA is a safe material for chemotherapy. A phase I report showed that intravenous injection of HMW-HA remained no harm and did not interfere with pharmacokinetics when combined with 5-fluorouracil and doxorubicin [170]. A phase IIa study illustrated using irinotecan enclosed by HA could extend the mean survival time and improve the tolerance in small cell lung cancer (SCLC) patients in extensive stages who carry CD44 positive [171]. Also, HA-coated irinotecan could prolong mean survival time and slightly extend the treatment response duration in metastatic colorectal cancer patients, compared with traditional 5-fluorouracil treatment [172]. The latest report indicates that based on the high affinity binding property of HA and CD44, the Fe_3_O_4_ nanocubes enclosed a small molecule inhibitor LDN193189 can be successfully delivered to HCC stem cells thus attenuating cellular proliferation, migration and EMT process [173]. The reason of HA coating system might be due to the increase in the potential for lymphotropic targeting to inhibit metastasis [174], as well as the high affinity towards HA receptors. The other reason might be good biocompatibility and safety of HA make it an ideal material to develop novel drug delivery systems.

Another prevalent application of HA is building hydrogel as a 3D scaffold to mimic the exact microenvironment. HA hydrogels containing a mixture of hyaluronan, collagen and matrigel could provide a similar ECM stiffness as mimicking the in vivo elasticity modulus of brain condition, which is further applying a better model for evaluating the malignancy of glioma [168]. With additional linkage reagents and other components of ECM, the model could repeat the ideal matrix for cultivating cells [175]. In chitosan-hyaluronan membrane-derived 3D culture models, the stemness phenotype is largely amplified as rapidly growing to tumor spheroids, increasing invasion and interstitialization. This application reproduces the in vivo growth and progression of tumor cells to a great extent [176].

However, one problem would be: no replenishment of HA normally generated by stromal cells to sustain dynamic balance in the model. Except that, the synthesis and hydrolysis of HA seesaw microenvironment, not only chemical components and biological progression had changed, but also the physical properties such as transit pH value, osmotic pressure, stiffness, and viscosity remained constantly fluctuated. Further modification could also concern adding a sponge system obsessing good mechano- or chemo- sensitivity into the HA hydrogel model so that the HA with specific molecule weight could be constantly pumped out when the microenvironment changes. Besides, HA hydrogels with unilateral cell strainer might achieve co-culture tumor cells with stromal cells, and easily re-built functional experiments of both tumor and stromal cells thereafter.

## Challenges and perspectives of HA application in the future

Despite the simple structure and components of naïve HA, its metabolism might be one of the most intricate bio-process. HA homeostasis displays the perfect interpretation of natural harmony that survival of the fittest only could help the organism own self-regulation, as many cells contained both HAS and HYALs. Additionally, HA metabolism structured a cycle whereby remodeling ECM matrix and energy supplement. However, more studies are required to understand the detailed proportion of HA polymers with different lengths, which might have great potential for therapeutic intention. If the ratio of HMW-HA to fgHA could be locked in the high value or occlude unresectable tumor vessel with undigestible HA coat, the tumor cells might be limited at in situ position, as well as lose the extracellular signal for invasive and distant migrated competence. Indeed, detecting serum concentration and a precise fragment of HA would provide a more meaningful diagnostic index, therapeutic modification, and prognosis predictor in certain diseases. However, due to the faster dynamic turnover and short half-life of HA in the circulation system, several challenges ought to be solved: how could researchers precisely capture circulated HA? When would be the best cut-off time for evaluating the activity of HA catabolism? And would it be possible to discover a safe tracer that could monitor HA turnover in vivo track?

In summary, due to the different molecular weights of HA components, HA shows dual characteristics in regulating tumor microenvironment. HA in ECM which is composed of HMW-HA, LMW-HA and fgHA, is attributed to the different catabolism yield of HA under certain circumstances. The ratio of the HA mixture does not stay invariant the same. In physiological condition, HMW-HA takes the biggest proportion while the LMW-HA becomes the major component in tumor ECM. HA mixture participates in the dynamic variation of the tumor microenvironment including conversion of the extracellular signal to intracellular signalling cascades, energy generation and consumption and remodeling microenvironment mechanical and biological properties, along with tumor progression. Additionally, the simple structure and good biocompatibility of HA allow for the industrial pipelining process and development of biomimetic materials application although there still are challenges present. From accurately tracing the origin and decomposition of circulated HA in vivo to precisely understanding the metabolism and regulation pathway of HA at a cellular level, cracking these challenges might solve the current dilemma in the knowledge of HA.

## Data Availability

Not applicable.
